# High Prevalence of Beijing and EAI4-VNM Genotypes among *M. tuberculosis* Isolates in Northern Vietnam: Sampling Effect, Rural and Urban Disparities

**DOI:** 10.1371/journal.pone.0045553

**Published:** 2012-09-24

**Authors:** Van Anh Thi Nguyen, Marc Choisy, Duy Hung Nguyen, Thanh Hoa Thi Tran, Kim Lien Thi Pham, Phuong Thao Thi Dinh, Jules Philippe, Thai Son Nguyen, Minh Ly Ho, Sang Van Tran, Anne-Laure Bañuls, Duc Anh Dang

**Affiliations:** 1 Department of Bacteriology, National Institute of Hygiene and Epidemiology, Hanoi, Vietnam; 2 MIVEGEC (IRD 224-CNRS 5290-Université Montpellier 1 et 2), Centre IRD, Montpellier, France; 3 Hung Yen Health Service, Hung Yen, Vietnam; 4 Department of Microbiology, Hospital 103, Military Medical University, Hanoi, Vietnam; 5 Department of Tuberculosis, Hanoi Medical University, Hanoi, Vietnam; St. Petersburg Pasteur Institute, Russian Federation

## Abstract

A total of 221 isolates of *M. tuberculosis* were sampled from hospitals and the general population in the northern plain of Vietnam, one of the most populated region of the country. Genotypic composition and diversity were characterized, and we investigated how they are affected by sampling (hospital vs. general population), correcting for potential confounding effects (location, age and gender of the patients). Spoligotyping and 12 MIRU-VNTR typing were used as first line. Then 15 MIRU-VNTR standard set was used, making 21 MIRU-VNTR typing for the clustered isolates. Result showed that 8 lineages and 13 sub-lineages were circulating in the region. The most predominant lineages were Beijing (38.5%) and EAI (38.5%). Others appeared with small proportions H (1.4%), LAM (1.8%), T (8.1%), X (0.9%), MANU (2.3%), and Zero (0.4%). Higher clustering rate was found in the hospital samples (17.9% in urban and 19.2% in rural areas) compared to the population ones (0%). The typical Vietnamese EAI4-VNM sub-lineage of EAI lineage accounted for 67% of EAI strains and was associated with older ages. Beijing genotypes were associated with younger, urban population and were characterized by high clustering rates. These characteristics strongly suggest that Beijing strains are invading the population, replacing the local EAI-VNM4, thus predicting a more serious tuberculosis situation in the future in the absence of more effective control strategies.

## Introduction

According to the World Health Organization (WHO) report in 2010, Vietnam ranked 12^th^ among the 22 highest tuberculosis (TB) burdened countries in the world and 3^rd^ after China and the Philippines in the western Pacific region [1], with 290,000 cases of all forms and 32,000 deaths reported in 2010 (WHO tuberculosis country profile). Although Vietnam was among the first few nations in Asia to achieve the WHO targets on case detection rate as well as direct observation treatment short-course (DOTS) coverage and cure rate (report from the National TB program in 1997 and WHO), there was no evidence of decrease in TB incidence during the following decade [2].

Extensive molecular studies on *M. tuberculosis*, the causative agent of TB, have revealed a high level of genetic diversity. Interestingly, the genotypes of the circulating strains vary greatly from population to population [3], [4], [5], [6], [7]. Such geographical differences are likely the result of both the history of *M. tuberculosis* spread and differences in population-specific transmission capacity of the different strains [4]. Molecular epidemiology studies and routine molecular typing are highly useful for TB control in a given area because they allow to identify recent transmission, populations at risk and risk factors for TB transmission [8].

In Vietnam, the estimated detection rate poorly reaches 54% (WHO tuberculosis country profile, 2009). The vast majority of the detected cases are hospital-registered patients. This indicates that a large proportion of patients would not be included in the samples of studies based only on hospital-registered patients. A bias is thus expected given that TB patients who come to hospitals generally do so because of severe symptoms. Since there are evidences for phenotypic differences (including virulence) in *M. tuberculosis* isolates of different genotypes [9], we can expect the genetic assemblage of the isolates from hospital patients to be different from those isolated from patients found by active case detection in the population.

In this study, we aimed at characterizing the genotypic composition and diversity of *M. tuberculosis* isolates in the northern plain of Vietnam and at investigating how they are affected by sampling. To this purpose, we used two sample sets collected from the northern plain of Red river delta, one of the most densely populated regions in Vietnam. The first one includes isolates passively collected from hospitals in Ha Noi and Ha Tay provinces, whereas the second one includes isolates from an active screening for TB patients in the general population in Hung Yen province. These provinces are proximate and defined according to the pre-2009 administrative divisions. In the analysis we corrected for a possible confounding location effect by categorizing Ha Noi province (the capital) as urban and the two other ones as rural. Other possible confounding factors, such as patients' age and gender were also accounted for in the study.

## Results

### Genotypic composition and diversity of *M. tuberculosis* strains in northern plain of Vietnam

A total of 221 *M. tuberculosis* isolates were analyzed by different typing schemes. Results are shown in table S1 and figure S1. The spoligotyping generated 53 spoligo patterns. Out of these 53 spoligo patterns, 38 patterns (200 isolates) were found in the SITVITWEB, and 15 patterns (21 isolates), designated as spoligotype nvn1 to nvn15, were not found in the database. The upper clades of the nvn spoligotypes were defined using SPOTCLUST and revised by MIRU-VNTR*plus*. All defined lineage/sub-lineage were determined with a probability of more than 0.99 except the clade of nvn5 with a probability of 0.97. All the other ones were designated as U (undesignated). Among the nvn spoligotypes, we found a cluster of 6 isolates, which were all found in the same district of Chuong My (Ha Tay province) and share the same new spoligo pattern nvn7. The nvn7 spoligotype was identified as belonging to EAI5 sub-lineage by SPOTCLUST (table 1).

**Table 1:** Spoligotypes not identified in the SITVITWEB database.

Spoligotyp designation^a^	Spoligo pattern	# (%)^b^	Lineage/sub-lineage^c^	Probability^d^
nvn1	▪▪▪▪▪▪□▪▪▪▪▪▪▪▪▪▪▪▪▪□▪▪□□▪▪▪▪▪▪▪□□□□▪▪▪▪▪▪▪	1 (0.5)	U	Low
nvn2	▪▪▪▪▪▪▪▪▪▪▪▪▪▪▪▪□□▪▪▪▪▪▪▪▪▪▪▪▪▪▪□□□□▪▪▪▪□▪▪	1 (0.5)	U	Low
nvn3	▪▪□▪▪▪▪▪▪□▪▪▪▪▪▪▪▪▪▪▪▪▪▪▪▪▪▪▪▪▪▪□□□□▪▪▪▪▪▪▪	1 (0.5)	T1	0.9999
nvn4	□□▪▪▪▪▪▪▪▪▪▪▪▪▪▪▪▪▪▪□□□□▪▪▪▪▪▪▪▪□□□□▪▪▪□□▪▪	1 (0.5)	LAM9	0.9997
nvn5	▪▪▪▪▪▪▪▪▪▪□▪▪▪▪▪▪▪▪▪▪▪▪▪▪▪▪▪□□□□□□▪▪▪▪▪▪▪▪▪	1 (0.5)	EAI5	0.9720
nvn6	▪▪▪▪▪▪▪▪▪▪▪▪□▪▪▪▪▪▪▪▪□▪▪▪▪▪▪□□□▪▪□▪▪▪▪▪▪▪▪▪	1 (0.5)	F33	1.0000
nvn7	▪▪▪▪▪▪▪▪▪▪▪▪□▪▪▪▪▪▪▪▪□▪▪▪▪▪▪□□□□▪□▪▪▪▪▪▪▪▪▪	6 (2.7)	EAI5	0.9916
nvn8	▪▪▪▪▪▪▪▪▪▪▪▪▪▪▪▪▪□□▪▪▪▪▪▪□□▪□□□□▪□▪▪▪▪▪▪▪▪▪	1 (0.5)	EAI4	0.9938
nvn9	▪▪□▪▪□□□□□□□□□□□□□□▪▪▪▪▪▪□□▪□□□□▪□▪▪▪▪▪▪▪▪▪	1 (0.5)	U	Low
nvn10	▪▪▪▪▪▪□□▪▪▪▪□□▪▪▪▪▪▪□□▪▪▪▪▪□□□□▪□□▪▪▪▪▪▪▪▪▪	1 (0.5)	U	Low
nvn11	□□□□□□□□□□□□□▪▪▪▪□▪▪▪▪▪▪▪▪▪▪▪▪▪▪□□□□▪▪▪□□□□	1 (0.5)	X3	0.9999
nvn12	□□▪▪▪▪▪▪▪▪▪▪□▪▪▪▪▪▪▪□▪▪▪▪▪▪▪□□□□▪□▪▪▪▪▪▪▪▪▪	1 (0.5)	EAI5	0.9970
nvn13	▪▪▪▪▪▪▪▪▪▪▪▪▪▪▪▪▪▪▪▪▪▪▪▪▪□□▪□□□□▪□▪▪▪□▪▪▪▪▪	2 (0.9)	EAI4	0.9938
nvn14	▪▪▪▪▪▪▪▪▪□▪▪▪▪▪▪▪▪▪▪▪▪▪▪▪□□▪□□□□▪□▪▪▪▪▪▪▪▪▪	1 (0.5)	EAI4	0.9887
nvn15	□▪▪□▪□□▪□▪□□□□□□□□□□□▪▪□□□□□□□□□□□□□▪▪▪▪▪□□	1(0.5)	U	Low

a Spoligotypes not identified in the SITVITWEB were assigned as “nvn” (stand for northern Vietnam), followed by a number for the differentiation of each individual spoligotype.

b Number and percentage out of the total studied samples.

c The lineage/sub-lineage was defined by SPOTCLUST.

d The probability of lineage definition by SPOTCLUST.

The spoligotyping of our sample revealed 8 lineages and 13 sub-lineages (table 2). The isolates of Beijing and EAI lineages were the most predominant, accounting for the same proportion of 38.5% (85 isolates) each. The remaining 23.0% of the total samples included Haarlem (1.3%), LAM (1.7%), MANU (2.2%), T (8.1%), X (0.8%), Zero (0.4%) and the U (undesignated) strains (8.5%). Among the EAI lineage, EAI4-VNM was the most prevalent sub-lineage accounting for 25.3% (56 isolates) of the total samples, followed by EAI5 (11.7%) and EAI1-SOM (1.4%) (table 2).

**Table 2:** Genotypic distribution of the studied *M. tuberculosis* strains.

			Hospital sample		Population sample	
Lineage	Sub lineage	Spoligotype	Urban # (%)	Rural # (%)	Rural # (%)	Total # (%)
**Beijing**	Beijing	1	33 (58.9)	30 (28.8)	19 (31.1)	82 (37.1)
	Beijing-Like	250	1 (1.8)		1 (1.6)	2 (0.9)
		2101	1(1.8)			1 (0.5)
**Total Beijing**			**35 (62.5)**	**30 (28.8)**	**20 (32.8)**	**85 (38.5)**
**EAI**	EAI1_SOM	1902		3 (2.9)		3 (1.4)
	EAI4_VNM	139	6 (10.7)	26 (25)	9 (14.8)	41 (18.6)
		564	1 (1.8)	1 (1)		2 (0.9)
		1731			1 (1.6)	1 (0.5)
		1901		1 (1)	1 (1.6)	2 (0.9)
		1903		3 (2.9)		3 (1.4)
		2450	1 (1.8)	1 (1)	1 (1.6)	3 (1.4)
		nvn	1 (1.8)		3 (4.9)	4(1.8)
	**Total EAI4-VNM**		**9 (16.1)**	**32 (30.8)**	**15 (24.6)**	**56 (25.3)**
	EAI5	152			6 (9.8)	6 (2.7)
		234	1 (1.8)			1 (0.5)
		236	2 (3.6)	3 (2.9)	2 (3.3)	7 (3.2)
		617			3 (4.9)	3 (1.4)
		618		1 (1)		1 (0.5)
		1372		1 (1)		1 (0.5)
		nvn		6 (5.8)	1(1.6)	7 (3.2)
**Total EAI**			**12 (21.4)**	**46 (44.3)**	**27 (44.3)**	**85 (38.5)**
**H**	H3	50	2 (3.6)		1 (1.6)	3 (1.3)
**LAM**	LAM9	42		2 (1.9)		2 (0.8)
	LAM4	60		1 (1)	1 (1.6)	2 (0.8)
**MANU**	MANU1	100		2 (1.9)	1 (1.6)	3 (1.3)
	MANU2	54		2 (1.9)		2 (0.9)
**T**	T1	53	3 (5.4)	3 (2.9)	1 (1.6)	7 (3.0)
		174		1 (1)		1 (0.5)
		334		2 (1.9)		2 (0.9)
		1067		1 (1)		1 (0.5)
		1105		1 (1)		1 (0.5)
		1560	1 (1.8)			1 (0.5)
		nvn		1 (1)		1 (0.5)
	T2	52			1 (1.6)	1 (0.5)
		1077			1 (1.6)	1 (0.5)
	T2–T3	73	1 (1.8)	1 (1)		2 (0.9)
**X**	X3	200			1 (1.6)	1 (0.4)
		nvn			1 (1.6)	1 (0.4)
**ZERO**		405			1 (1.6)	1 (0.4)
**U**		124			1 (1.6)	1 (0.5)
		458	1 (1.8)	2 (1.9)	1 (1.6)	4 (1.8)
		523		1 (1)		1 (0.5)
		623		3 (2.9)	1 (1.6)	4 (1.8)
		1904		1 (1)		1 (0.5)
		2379			1 (1.6)	1 (0.5)
		nvn	1 (1.8)	5 (4.8)	1 (1.6)	7 (3.0)
**Total**			**56 (100)**	**104 (100)**	**61 (100)**	**221 (100)**

Twenty-one clusters (189 isolates) and 32 unique isolates were identified by spoligotyping. Among these clusters, the most frequent spoligotypes were the international types (SIT)_1 (Beijing), 37.1% (82 isolates), and SIT139 (EAI4-VNM), 18.6% (41 isolates). The other 15 clustered spoligotypes were shared by 2–7 isolates (table 2).

The 12 MIRU-VNTR typing further divided the 21 spoligo-clusters into 113 MIRU-VNTR profiles including 21 clusters (97 isolates) and 92 unique profiles. The clustering rate was 34.4%. Among the 21 clusters, there were 7 large clusters shared by 6 EAI5 isolates from Chuong My, 18, 17, 9 and 5 Beijing isolates, and 7 and 4 EIA4-VNM isolates. The next 14 clusters were small, shared by 2 isolates, excepted 2 clusters by 3 isolates.

The 97 clustered isolates determined by 12 MIRU-VNTR locus set were then typed by 15 MIRU-VNTR set (i.e. 9 additional loci were applied resulting in genotyping by 21 loci in total). Sixty-one MIRU-VNTR profiles including 16 clusters (52 isolates) and 45 unique profiles were generated. The clustering rate reduced to 16.3%. The sizes of the large Beijing clusters reduced to 10, 3, 2, 7 and 2 isolates, and those of the large EAI4-VNM clusters reduced to 2 and 3. Of the 14 small clusters, 9 remained the same, the other 5 were further divided into unique patterns. The isolates in each cluster of all the 14 small clusters of 2–3 isolates, identified after 15-MIRU-VNTR typing, were consistently found to be from patients living in the same district. All the 6 isolates from Chuong My district, sharing spoligotype nvn7, had identical 12- and 15-MIRU-VNTR profiles and were different from the MIRU types of all other strains that were typed. The 12-MIRU type of the isolates was named Mnvn26 (table S2).

The 12-MIRU typing identified respectively 33, 34 and 45 MIRU types for the strains belonging to Beijing and EAI4-VNM lineage/sub-lineage and the strains other than Beijing and EAI4-VNM, of which 13, 9 and 13 types were found in the SITVITWEB database or previously identified and 20, 26 and 32 types were not found, respectively (table S2). Sixty-seven percent of the urban isolates had international MIRU types (MITs), while only 35% of the rural isolates had MITs. Among the latter, the percentage of MITs was lower in the population samples (20%) than in the hospital samples (42.7%). The differences were significant (*p*<0.001).

**Figure 1:**
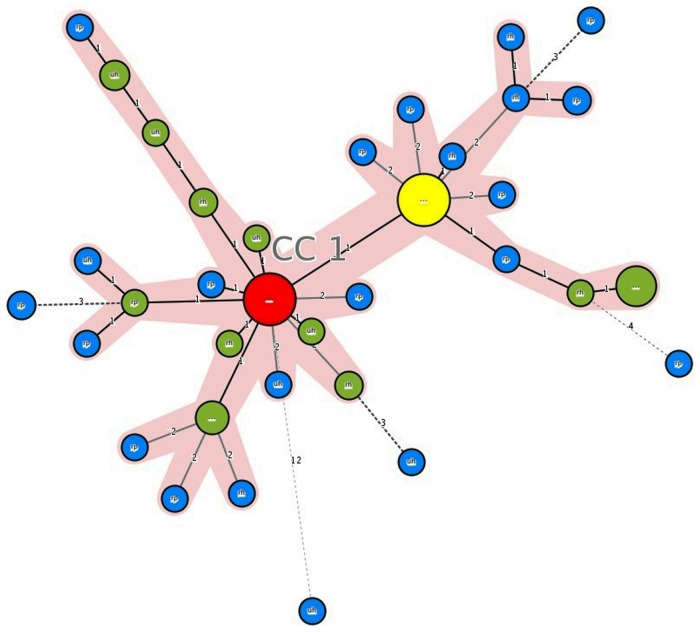
Minimum spanning tree of Beijing isolates based on 12-MIRU types. Red = M11, yellow = M33, green = MIT, blue = B-Mnvn. The sizes of the circles correspond to the frequencies of the MIRU types. The numbers on the connection lines indicate the numbers of locus differences.

**Figure 2:**
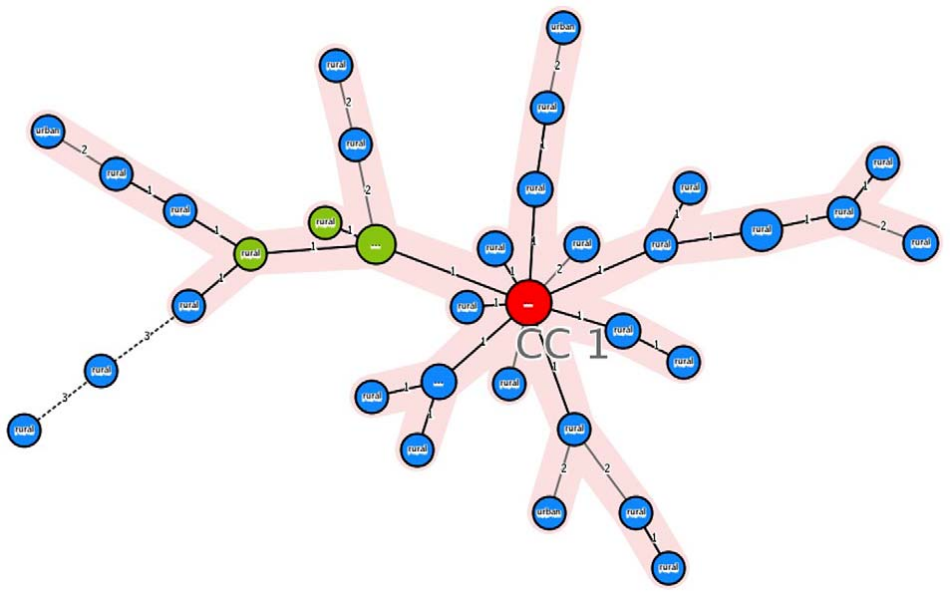
Minimum spanning tree of EAI4-VNM isolates based on 12-MIRU types. Red = MIT58, green = MIT, blue = EAI4-Mnvn, yellow = ORPHAN in SITVITWEB. The sizes of the circles correspond to the frequencies of the MIRU types. The numbers on the connection lines indicate the numbers of locus differences.

Minimum spanning tree of the 12-MIRU types of Beijing isolates shows the core position of the two previously identified types M11 (MIT17) and M33 (MIT83). The majority of MIRU types found in SITVITWEB or previously identified are linked to M11, while the majority of MIRU types linked to M33 were not identified previously (B-Mnvn) (figure 1). Further typing using 15 MIRU loci revealed that two genotypes generated from M11 and the genotype remaining in the big cluster generated from M33 are the core genotypes (i.e. remain in the core position), forming 3 different branches in the minimum spanning tree (table S2, figure not shown). The minimum spanning tree of the 12-MIRU types of the EAI4-VNM isolates also shows a core position of MIT58 (figure 2). Further typing by 15 MIRU loci revealed the core position of one of the genotypes generated from MIT58 (table S2, minimum spanning tree created by 15-MIRU typing not shown).

**Figure 3:**
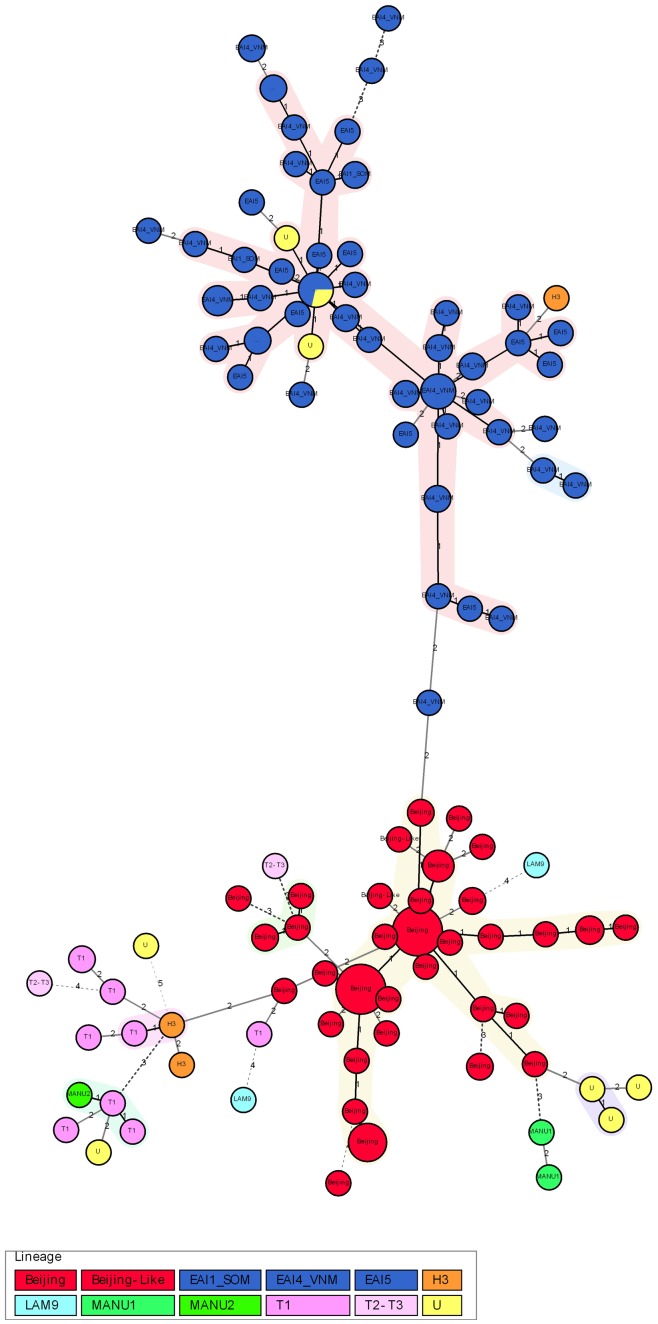
Minimum spanning tree of the studied *M. tuberculosis* isolates in the northern Vietnam based on 12-MIRU types. Blue = EAI lineage, red = Beijing lineage. The sizes of the circles correspond to the frequencies of the MIRU types. The numbers on the connection lines indicate the numbers of locus differences.

All strains belonging to the two main lineages Beijing and EAI could be differentiated from each other by MIRU24 (1 repeat for Beijing/2 repeat for EAI, sensitivity 100%, specificity 100%) or MIRU26 (1 and 4–10 repeat for Beijing/2–3 repeat for EAI, sensitivity 100%, specificity 100%). Strains belonging to these two lineages were separately aggregated into two main branches of the minimum spanning tree, based on 12 MIRU-VNTR typing (figure 3). The sensitivity and specificity for the identification of Beijing genotypes were respectively 100% and 76.3% by MIRU24 and were 100% and 80.7% by MIRU26. The sensitivity and specificity for the identification of EAI genotypes were respectively 100% and 95.3% by MIRU24 and were 100% and 91.7% by MIRU26. The combination of the two MIRU loci does not increase the sensitivity and specificity for the identification of the two lineages (table 3).

**Table 3:** Sensitivity and specificity for the differentiation and identification of Beijing and EAI lineages by MIRU24 and MIRU26.

MIRU	Repeat	Differentiation between Beijing and EAI lineages		Identification of Beijing lineage		Identification of EAI lineage	
		Se (%)	Sp (%)	Se (%)	Sp (%)	Se (%)	Sp (%)
MIRU24	1	100	100	100	76.3		
	2	100	100			100	95.3
MIRU26	1, 4–10	100	100	100	80.7		
	2, 3	100	100			100	91.7
MIRU24 & MIRU26	1 & 1, 4–10	100	100	100	80.7		
MIRU24 & MIRU26	2 & 2, 3	100	100			100	95.3

### Sampling effect on the genotypic composition and diversity of *M. tuberculosis* strains

**Figure 4:**
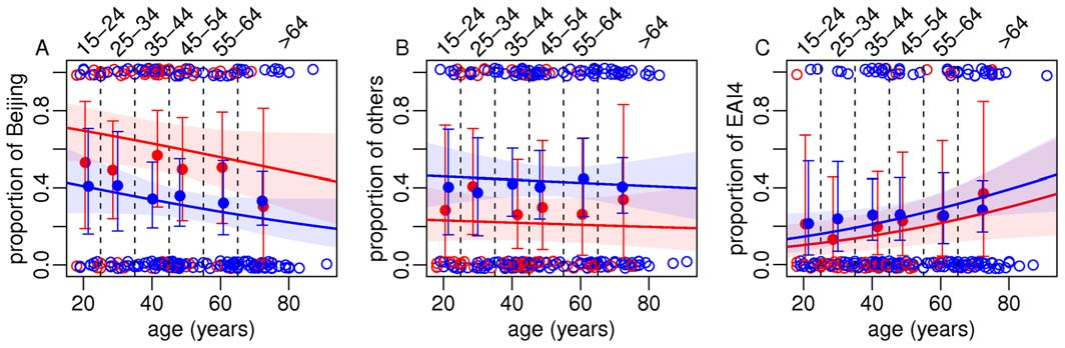
Proportion of Beijing (A), EAI4-VNM (C) isolates and others (B) as functions of age and location (urban in red vs. rural in blue). Empty circles represent raw data (1 and 0 for yes and no), filled dots are the means defined for each age class (represented by vertical dotted bars and labeled on the top of the graph), together with 95% confidence interval (vertical bars). The lines are the logistic model predictions (see table 5 for the parameter estimates and their significativities) together with 95% CI (shaded area).

**Figure 5:**
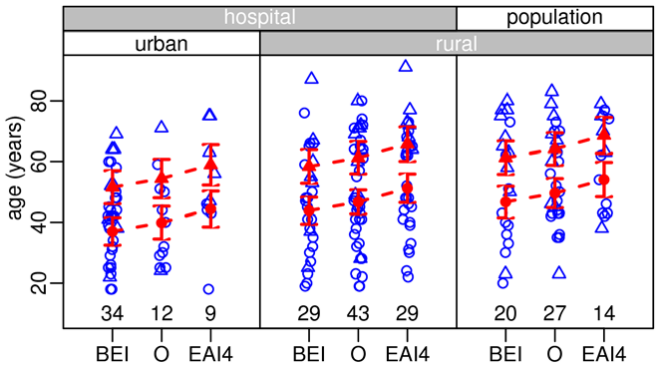
Patient's age as a function of genotypes (Beijing [BEI], EAI4-VNM [EAI4], and others [O]) genotype, location (urban vs. rural), sampling (hospital vs. population), and sex (triangle for female, circle for males). Blue open triangles and circles are the row data whereas red filled triangles and circles are the analyses of variance predictions, together with 95% confidence intervals (vertical lines).

Logistic regression analyses showed no significant effect of sampling on the proportion of Beijing and EAI4-VNM isolates (tables 4 and 5) but showed a significant higher proportion of clustered isolates in the hospital samples than in the population samples (no cluster was found in the population samples) (*p*<0.001, table 6). Furthermore, the proportion of clustered isolates was significantly higher among Beijing strains than among the other strains (*p*<0.001, table 6). The analyses also showed that there was a significant association between the proportion of Beijing isolates and patients' age (higher among the younger age classes) (*p*<0.05). The proportion of Beijing isolates was higher in urban districts than in rural ones (*p*<0.001) (table 4 and figure 4). The proportion of EAI4-VNM, on the contrary, significantly increased with age (*p*<0.05) (table 5 and figure 5). These results are clearly visible on figure 5 and table 7, which also show that female patients were older than male ones (*p*<0.0001). Finally, clustering rates (CRs) were higher in the hospital isolates (urban: 17.9%, rural: 19.2%) than in the population isolates (0%). The CRs were 25.9%, 10.7% and 10.0% for the isolates of Beijing, EAI4-VNM and other genotypes respectively.

**Table 4:** Analysis of deviance analysis table of logistic regression of proportion Beijing isolates as a function of age and sex of the patient, location (urban vs. rural), and sampling (hospital vs. population).

	Estimate*	Df	Deviance*	*p*(>|χ^2^|)*
(Intercept)	0.6631			
age	−0.0205	1	4.4320	0.0353
sex (male)	−0.4357	1	1.4677	0.2257
sampling (hospital)	−0.2257	1	0.3912	0.5317
location (urban)	1.2469	1	12.3167	0.0004

* : Estimates, deviance and their *p*-values are calculated by correcting for possible confounding effects of all the other explanatory variables. (see Materials and methods).

**Table 5:** Analysis of deviance table of logistic regression of proportion of EAI4-VNM isolates as a function of age and sex of the patient, location (urban vs. rural), and sampling (hospital vs. population).

	Estimate*	Df	Deviance*	*p*(>|χ^2^|)*
(Intercept)	−2.8132			
age	0.0259	1	5.7873	0.0161
sex (male)	0.2035	1	2.2669	0.6055
sampling (hospital)	0.4011	1	1.0975	0.2948
location (urban)	−0.5177	1	1.4401	0.2301

* : Estimates, deviance and their *p*-values are calculated by correcting for possible confounding effects of all the other explanatory variables. (see Materials and methods).

**Table 6:** Analysis of deviance table of the logistic regression of the proportion of isolates in clusters as a function of genotype, sex and age of the patient, location (urban vs. rural), and sampling (hospital vs. population).

	Estimate*	Df	Deviance*	*p*(>|Chi|)*
(Intercept)	−19.6007			
age	−0.0103	1	0.5783	0.4470
Sex (male)	−0.1162	1	0.0507	0.8218
Strain (Beijing)	1.4458	1	10.9667	0.0009
Strain (EAI4-VNM)	0.0926	1	1.9483	0.1628
location (urban)	−0.5619	1	1.5603	0.2116
sampling (hospital)	18.3649	1	23.6404	<0.0001

* : Estimates, deviance and their *p*-values are calculated by correcting for possible confounding effects of all the other explanatory variables. (see Materials and methods).

**Table 7:** Analysis of variance table of the patients' age as a function of genotype (Beijing, EAI4), sex of the patient, location (urban vs. rural), and sampling (hospital vs. population).

	Estimate*	Df	Deviance*	*p*(>|χ^2^|)*
(Intercept)	64.087221			
sex (male)	−14.539384	1	9106.5446	<0.0001
Beijing (vs. non Beijing)	1010.1789	1	4.3628	0.0379
Beijing (vs. others)	−2.8642	1	313.7957	0.2437
EAI4-VNM (vs. non EAI4-VNM)	1335.6824	1	5.8071	0.0168
EAI4-VNM (vs. others)	4.4943	1	639.3010	0.0967
location (urban)	−6.8148	1	1525.6521	0.0106
sampling (hospital)	−2.8813	1	307.1028	0.2488

* : Estimates, deviance and their *p*-values are calculated by correcting for possible confounding effects of all the other explanatory variables. (see Materials and methods). We applied two different contrasts to test the effects of Beijing and EAI4-VNM genotypes.

## Discussion

Our 221 isolates were identified to belong to 53 spoligotypes. Nearly 28% (15/53) of the spoligotypes and 69% (77/112) of the 12-MIRU types characterized in the present study were absent from the recently updated SITVITWEB database [10], which suggests that a large number of genotypes in northern Vietnam are still not identified. The spoligotypes belonged to 8 lineages and 13 sub-lineages. Among these, the two most predominant lineages were Beijing and EAI. Other lineages appeared with small proportions. The general picture coincides with the situation of *M. tuberculosis* lineage distribution in East Asia [7]. However, specific features of Beijing and EAI lineages make the tuberculosis epidemiology in Vietnam singular as detailed below.

### Recent invasion of Beijing lineage in Vietnam

The proportion of Beijing genotypes in our study was not as high as in other Asian countries: 58% in Thailand, 69% in Hong Kong, 77% in Korea, 80% in Japan, and 82% in China [11], [12], [13], [14], [15]. In a previous study based on samples from a nation-wide survey on drug resistant TB in Vietnam in 2005, the overall proportion of Beijing isolates was 35% (30% in the North, 28% in the Center and 39% in the South) [16]. The seemingly higher proportion of Beijing isolates found in our study (38.5%, to compare with the above-cited 30% for the North) could be explained by the fact that our samples came from the most densely populated area in northern Vietnam. Indeed, our study clearly shows a higher proportion of Beijing isolates in urban than in rural areas (62.5% vs. 28.8%, *p*<0.001). A high percentage of Beijing isolates (54%) was also previously reported among *M. tuberculosis* isolates collected from two reference hospitals in the two biggest Vietnamese cities of Hanoi and Ho Chi Minh in 1998–1999. Moreover, we found that Beijing genotypes were associated with younger age, as expected for recently introduced pathogens. This finding is in accordance with other studies in Vietnam, including the one carried out in a rural area in southern Vietnam [17], [18]. Association between the proportion of Beijing strains and patients' age does not exist in most Asian countries, except Bangladesh and Hong Kong [5], [19] showing similar and opposite trends respectively. [17].

To explain the location effect on the time trend and the proportion of Beijing isolates we hypothesize that Beijing strains were recently introduced to Vietnamese urban areas by international human migrations and travels, from where they spread to rural areas of Vietnam. Such a hypothesis is in accordance with Buu et al. (2009) who found infections with Beijing genotypes more common in patients living along the main road to Ho Chi Minh city in their studied rural population of southern Vietnam [18]. This hypothesis is further comforted by the fact that there was a significant higher proportion (67%) of studied isolates having international MIRU types (identified in SITVITWEB) in urban areas than in rural areas (35%). The 12-MIRU types of all large clusters of Beijing isolates in our sample were found to be highly prevalent in many other East Asian countries such as Wuhan-China, Taipei, Singapore, Hong Kong [20], Japan, Mongolia [15], and Russia [21], where Beijing strains were predominant for many decades with no time trend [5]. Among them, a previously suggested ancestral Beijing MIRU type (M11) [21], [22], one of the most prevalent genotypes in other countries in the region [15], [20], [21] was also found one of the most prevalent Beijing-MIRU types in our study, accounting for 19% of the total Beijing strains. The most prevalent Beijing-MIRU type in this study was M33 (21% of the total Beijing ones). The high prevalence of this genotype in Vietnam was previously reported by Mokrousov et al. (2005) when analyzing the Beijing isolates in one part of our samples [22]. This genotype was also found to be the most prevalent Beijing genotype in Shanghai (China), South Vietnam and to lesser extent in Singapore, Hong Kong and Japan [23]. The hypothesis that Beijing strains originated from Central Asia and spread to East Asia during Neolithic Age [22] and the unique high prevalence of M33 genotype along coastal regions of East and Southeast Asia, especially in Shanghai, compared to other parts of Asia [23] suggest that the sea route may have played a role in the spread of the M33 genotype (especially from Shanghai where M33 seems to be the local specific strain) to other East and Southeast Asian countries including Vietnam. The regional specificity of M33 genotype in East and Southeast region is supported by our finding that very few international MIRU types found in our samples are linked to this genotype, the majority of them being linked to M11 instead. In the minimum spanning tree, M11 and M33 are linked to each other and are in the core position, forming the two main branches of the system (figure 1). Since it is suggested that *M. tuberculosis* evolves by loosing rather than gaining repeats [24], M33 seems to be derived from the ancient genotype M11 by the lost of 1 repeat at MIRU 26 (from 7 to 6). The fact that all genotypes linked directly to M33 were local previously unidentified suggests that the M33 could have been imported to Vietnam early in time. Thus, the genetic composition of the M33 branch in the minimum spanning tree would be a result of a local evolution. On the other hand, 15-MIRU typing revealed that the majority of genotypes sharing M11 type are unique, with only 5 isolates in 2 small clusters. There are two genotypes generated from M11 remaining in core position, one is unique and the other one is clustered. The 2 core genotypes are different from each other by 4 MIRU loci (table S2), forming 2 far-distanced branches. This indicates that strains sharing genotype M11 in our study are very diverse and seem to be a result of recent importation since they are linked to the majority of the international MIRU types found in this study (figure not shown). Finally, our finding that Beijing genotypes had higher clustering rate compared to others suggests a high transmission potential for Beijing strains, comforting again the hypothesis that Beijing is an invasive lineage in Vietnam.

### EAI4-VNM is a locally specific lineage in Vietnam

The Vietnamese genotypes EAI4-VNM, a sub-lineage of EAI lineage [7] accounted for 67% of the total EAI isolates or 25.3% of the total samples. In a recent study in 3 rural districts of southern Vietnam, where hospital sampling was used, EAI4-VNM genotypes were found in 50.7% of the total *M. tuberculosis* isolates (compared to 30.8% in rural hospital samples in the present study) [25]. This indicates a large variation in the proportion of EAI4-VNM isolates in different regions of Vietnam. The proportion of Beijing isolates seems to be less variable (28.8% in the rural North as shown this study and 32.5% in the rural South as mentioned in the above study, in hospital samples). EAI4-VNM genotypes were much less frequent in other nations as shown in the SITVITWEB database and even the neighboring countries: Thailand (3 out of 152 meningitis TB cases) [11], Cambodia (6 out of 105 TB cases) [26], Indonesia (0 out of 897 TB cases) [27], Myanmar (0 out of 310 TB cases) [28]. The fact that EAI4-VNM isolates are numerous only in Vietnam, especially in rural areas of South Vietnam [25] but not elsewhere, suggests that the strains may have originally appeared in South Vietnam. This is probably a result of the settlement of the previously suggested ancestral EAI strains when it spread out of the African continent to East Asia, concomitantly with ancient human migration [4], [7], [29]. This also suggests a more specific adaptation of this family to the local population. In fact, almost all EAI4-VNM isolates found in the SITVITWEB database with information on the origin are either from Vietnam, or from patients originated from Vietnam, or from countries with large Vietnamese populations such as USA.

Our finding that the proportion of EAI4-VNM isolates increases with the age of the patients leads to the hypothesis that EAI4-VNM strains of *M. tuberculosis* may have appeared in Vietnam earlier than the recently introduced Beijing strains or to the alternative hypothesis that EAI4-VNM strains are more prone to the elimination by the herd immunity gained from the introduction of mass-BCG vaccination in Vietnam. If the latter hypothesis is true, the vaccine seems to fail to protect adequately the young people infected with Beijing strains (Beijing genotypes associated with younger age groups). However, the former hypothesis is more likely because BCG vaccination has been applied in Vietnam for only 27 years since 1985 (Vietnam National Expanded Immunization Program: http://www.nihe.org.vn/new-en/chuong-trinh-tiem-chung-mo-rong-quoc-gia-703261429/1063/Contents.vhtm), or barely 20 years at the time of the collection of the studied samples. Thus, the association of the proportions of Beijing and EAI4-VNM isolates with ages should not be seen in patients older than 20. However, the association was observed in all age groups (as shown by logistic regression analysis, figure 2). In a previous study investigating the correlation between drug resistance and BCG vaccination with typical and atypical Beijing sub-lineages both in Vietnam and the Netherlands, a significantly higher proportion of typical Beijing isolates was found in the vaccinated group compared to the non-vaccinated group in the samples from the Netherlands, but not in the samples from Vietnam [30].

### Higher clustering rate in the hospital isolates

The standardized MIRU–VNTR typing using the 15 and 24 loci sets was proved to be useful for population-based epidemiological studies and for studies on the evolution and transmission of *M. tuberculosis* lineages in populations, including populations predominated by Beijing family strains [15], [31]. In this study, both standardized 12 and 15 MIRU locus sets were used for the typing of clustered isolates, thus 21 MIRUs were used in total when accounting for the common 6 loci to the two sets. The MIRUs in the full 24 MIRU set that have not been used for the typing are Mtub29, ETRB, Mtub34. In the previous study, these MIRUs had low Hunter-Gaston discriminatory indexes (HGI) of 0.119, 0.014 and 0.014 for Beijing isolates and the subtle difference in HGIs between 15- and 24-MIRU typing schemes was only due to Mtub29 [32]. The clustering rate of our sample is thus expected not to be much affected by the use of 24-MIRU typing scheme. In the above-mentioned study, the IS*6110* restriction fragment length polymorphism (IS*6110*-RFLP) (HGI, 0.999) was superior to the 24-MIRU typing (HGI, 0.992) and the 15-MIRU typing (HGI, 0.990) when used for the typing of the Beijing isolates from Beijing, China [32]. However, the interpretation of IS*6110*-RFLP profiles for epidemiological investigations should be performed with caution since IS*6110* pattern changes were found in 16% of the *M. tuberculosis* isolates from patients in actual chains of transmission in a study from Iran [33]. IS*6110*-RFLP typing can thus lead to an underestimation of molecular clustering rates. In our study, we found no cluster among the 30 Beijing isolates in the population samples, while in the majority of the clustered isolates (43/52) and of the clustered Beijing ones (26/29) in the hospital samples, epidemiological links (patients living in the same districts) were observed. Moreover, the MIRU types of the large Beijing clusters are the types that largely predominate in many other countries (as shown in SITVITWEB database and by Mokrousov et al. [23]) regardless of the possible differences in host-pathogen interactions. This indicates that these genotypes may be highly transmissible. Thus, the high clustering rates in the hospital samples were certainly due to recent transmissions rather than to poor discriminatory powers of the typing schemes.

The higher clustering rates in the hospital samples than in the population samples is in accordance with the trade-off hypothesis on virulence predicting that the strains with the highest transmission capacity would also be the ones producing the most severe symptoms (that push the patients to seek care in hospitals) [34]. In this study, the international genotypes were found with a significant higher proportion (42.7%) in the hospital isolates compared to the population ones (20%) (correcting for the potential confounding location effect). This indicates that the international genotypes are more virulent than the local previously unidentified ones. Another possible cause for the higher clustering rate in the hospital samples could be the long treatment delays that often happened to TB patients. Such delays are in general due to the chronic nature of the disease. For Vietnamese patients in particular, the delays can also be due to the fact that TB is not only a cause of social stigma and social isolation but also a cause of increasing poverty through job losing and health care expenses in and thus patients do not seek for health care before the symptoms become much advanced [35]. Such a delay in health check might lead to higher transmission in their communities through daily contacts. This possibility is supported by the fact that epidemiological links (living in the same districts) were found for the majority of the clustered isolates. Previous studies reported that the mean delay for TB diagnosis in TB patients in Vietnam was 12 weeks while the recommendation is less than 3 weeks [36].

### Current transmission of a new genotype in Chuong My, Ha Tay

The 6 isolates in Chuong My, Ha Tay sharing the same unique spoligotype nvn7 and the same unique 12- and 15-MIRU types, could have originated from a single isolate. Their MIRU type was different to that of any other strains that were typed in our study. The high number of cases caused by this genotype in the same district may reflect a possible TB outbreak caused by this strain in the area.

### MIRU-VNTR typing as routine molecular typing in Vietnam

The spanning tree created by MIRU-VNTR*plus* based on 12 MIRU-VNTR typing showed a distinct separation of Beijing and EAI lineages from each other. The two lineages can be differentiated from each other with the sensitivity and specificity of 100% and 100% respectively, by either MIRU24 or MIRU26. The sensitivity for the identification of these two lineages by either MIRU24 or MIRU26 was also 100%. The specificity for the identification of these two lineages achieved by MIRU24 and MIRU26 were 76.3% and 80.7% for Beijing isolates and 95.3% and 91.7% for EAI isolates, respectively. It is lower for Beijing due to the genotypic similarity of these strains to the strains other than Beijing and EAI. In the minimum spanning tree, there are a number of other strains also locating in the Beijing branch. However, the Beijing strains are more aggregated in the center of the branch and connect to each other through links of 1 or 2 locus differences, while other strains (except some U strains) are more scattered at the end of the Beijing branch, mostly connected to the Beijing strains through links of 3 or more locus differences (more genotypically different) (figure 3). This finding suggests that MIRU-VNTR typing was congruent with spoligotyping and can be used to determine Beijing and EAI lineages even without the help of spoligotyping. The identification can be done with the use of the previously identified strains as references. This advantage would be of great help to promote routine molecular typing for *M. tuberculosis* strains in low-income countries where Beijing and EAI lineages are predominant like Vietnam. There were a number of U strains embedded in the two Beijing and EAI branches of the minimum-spanning tree. This finding suggests that these unidentified strains could belong to these two lineages and that MIRU typing might help to determine the strains that cannot be identified by spoligotyping. Other typing methods such as SNP typing should be used for the confirmation of this hypothesis.

Finally, our results confirm the largely documented fact that males are more affected than female by TB (71% vs. 29%) [37]. Logistic regression analysis showed no significant effect of sampling method (population vs. hospital), region (urban vs. rural) and lineage (Beijing and EAI) on gender (table 4 and 5). Female patients are older than male patients and TB patients in urban areas are younger than in rural areas.

In summary, our observations strongly suggest that Beijing lineage is currently invading Vietnam. While the local EAI4-VNM predominant genotypes have been found to be the most drug susceptible strains in Vietnam (unpublished data), Beijing genotypes have been documented to be associated with drug and multi-drug resistance in Vietnam and elsewhere [18], [19], [38], [39]. This invasion and replacement would predict a more serious TB situation in Vietnam in the future in absence of more effective control strategies.

## Materials and Methods

### Ethics Statement

Written informed consents were obtained from study participants. All study procedures were approved by ethical review committees at the National Institute of Hygiene and Epidemiology, Vietnam.

### Strain collections

Two collections of *M. tuberculosis* isolates were used in this study (figure S2). The first one includes 160 isolates from a hospital-based sampling. Over a period of one year from December 2003 to December 2004, the *M. tuberculosis* isolates were from all newly diagnosed acid fast bacillus (AFB) smear positive patients in 8 district TB prevention centers randomly selected among a total of 22 in the two provinces of Ha Noi and Ha Tay. Out of these 8 studied districts, two were categorized as urban since they are located in the center of the capital Ha Noi (56 isolates), the other 6, including suburban and rural area, were categorized as rural (104 isolates). The second collection included 61 *M. tuberculosis* isolates from a population-based sampling carried out from May to November 2005, in Hung Yen province. Following the same rationale as before, this province was categorized as rural. Stratified sampling was applied for the selection of districts and cluster sampling was used for the selection of the households investigated. The screened subjects were all people having cough for more than 2 weeks. They were then examined by sputum smear microscopy. Upon positive result for AFB, another sputum sample was collected for isolation of the bacillus.

### Molecular typing

The isolates were typed with a 43-spacers membrane, as previously described [40], [41] and then entered in the recently published SITVITWEB (http://www.pasteur-guadeloupe.fr:8081/SITVIT_ONLINE/) in order to identify shared types and phylogenetic lineages [10]. For the spoligotypes not found in the SITVITWEB database, their spoligotypes were assigned as “nvn” (standing for northern Vietnam) followed by a number for the differentiation of each individual spoligotype and their lineage/sub-lineage were defined using SPOTCLUST (http://tbinsight.cs.rpi.edu/run_spotclust.html) [42] and revised by MIRU-VNTR*plus* [43]. Since many studies carried out in East Asian region were based on the 12 international standardized MIRU-VNTR loci for typing *M. tuberculosis* isolates [15], [20], [21], [23], in order to compare with these studies, we also used the 12 locus set to further type the isolates identified in clusters by spoligotyping (189 isolates) [44]. Since the new 15 standardized MIRU-VNTR set which shared 6 MIRU-VNTR loci with the old 12 MIRU-VNTR set was proved to have greater discriminatory power over the 12 one [45], it was used to type the isolates remained in clusters by the 12 MIRU-VNTR typing (97 isolates). Thus, the clustered isolates were finally identified by spoligotyping and 21 MIRU loci in total. The methods for MIRU-VNTR typing and the MIRU-VNTR loci were used as described in previous studies [44], [45]. The 12-MIRU types of the studied isolates were entered into the SITVITWEB to identify international MIRU types (MIT) and were compared with MIRU type (M) previously identified by Mokrousov et al. [21], [23]. For the MIRU types not found, their MIRU types were assigned as “B-Mnvn, EAI4-Mnvn” (standing for Beijing and EAI4-VNM-MIRU type in northern Vietnam, respectively), followed by a number for the differentiation of each individual type. The MIRU type of the strains other than Beijing and EAI4-VNM were assigned as Mnvn, followed by a number for the same reason.

### Data analyses

The sensitivity (Se) and specificity (Sp) for the differentiation or identification of strains belonging to a lineage by specific repeats of certain MIRU(s) were calculated as follows: Se = a/(a+c), Sp = b/(b+d) where a is the number of isolates belonging to the lineage identified by the specific repeats of the MIRU(s), c is the number of isolates belonging to the lineage not identified by the specific repeats of the MIRU(s), b is the number of isolates that do not belong to the lineage not identified by the specific repeats of the MIRU(s), d is the number of isolates that do not belong to the lineage identified by the specific repeats of the MIRU(s) [46]. A molecular cluster was defined as a cluster of two or more isolates having identical genotypes by both spoligotyping and MIRU-VNTR typing. Recent transmission can be approximated by the clustering rate (CR) [47]. CR = (n_c_−c)/n where n_c_ is the total number of clustered isolates, c is the total number of clusters, and n is the total number of isolates [48]. Since there is one case in each cluster that should be considered as the primary case, we reduced the number of isolates in each cluster by one when analyzing the proportion of cluster isolates of different genotypes. Minimum spanning trees were created using MIRU-VNTR*plus* (http://www.miru-vntrplus.org/) [43]. Logistic regression (with logit links) were used to investigate how (i) age, (ii) sex of the patient, (iii) location (urban versus rural), and (iv) sampling strategy (population-based versus hospital-based) can affect (i) the proportion of isolates of different genotypes and (ii) the proportion of clustered isolates of different genotypes. In our study, isolates of Beijing and EAI4-VNM genotypes were, by far, the most prevalent in the studied region and EAI4-VNM is the typical genotype for Vietnam. Consequently, in the analyses on the proportion of isolates of different genotypes, we considered three groups: Beijing, EAI4-VNM, and others (i.e. other than Beijing and EAI4-VNM). In the calculation of *p*-values we correct for possible confounding effect due to colinearities between the explanatory variable by calculating type-III deviances [49]. Finally, we used analysis of variances to investigate how age was affected by (i) sex of the patient, (ii) location, (iii) sampling strategy, and (iv) genotype. Type-III sums squares were used in order to correct for possible confounding effect due to colinearities between the explanatory variables [49]. All two-by-two interactions were considered but none of them appeared to be significant. They were thus systematically removed from the models presented in the result section. All the statistical analyses were performed with the R software (R Development Core Team, 2010).

## Supporting Information

Figure S1 **Molecular typing schemes and results.**
(PDF)

Figure S2 **The collections of** ***M. tuberculosis*** **isolates in the northern Vietnam.**
(PDF)

Table S1 ***M. tuberculosis*** **isolates in the northern Vietnam (separate file).**
(XLS)

Table S2 **MIRU types of Beijing, EAI4-VNM strains and the strains other than Beijing and EAI4-VNM from northern Vietnam (separate file).**
(DOC)
