# Supplementary figures and images for: High Prevalence of Beijing and EAI4-VNM Genotypes among M. tuberculosis Isolates in Northern Vietnam: Sampling Effect, Rural and Urban Disparities
Source: PLoS One. 2012 Sep 24;7(9):e45553. doi: 10.1371/journal.pone.0045553 (PMC3454422; doi:10.1371/journal.pone.0045553)

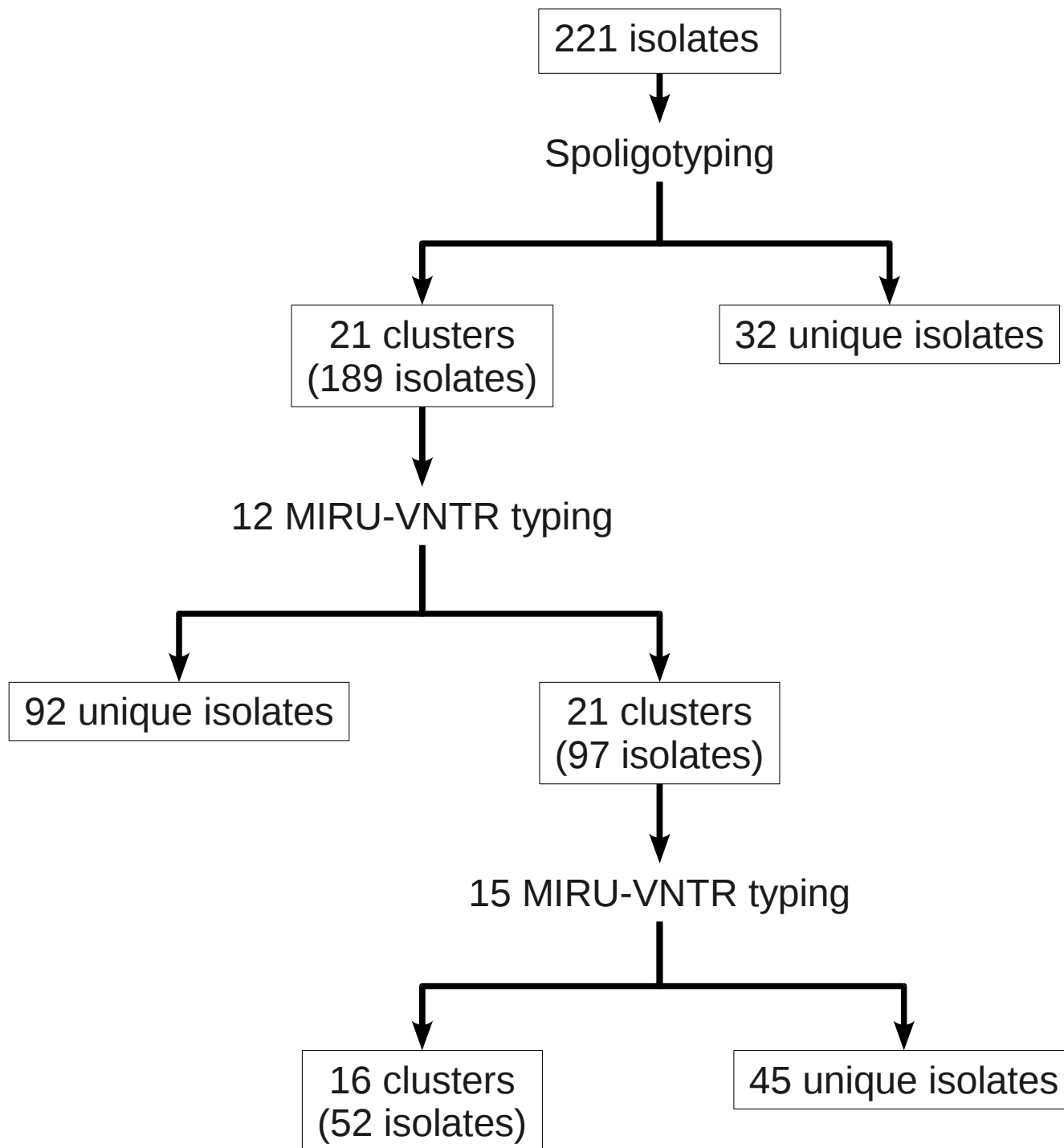

Supplement: Figure S1 — Molecular typing schemes and results. (PDF) [file pone.0045553.s001.pdf]
