# Supplementary material for: High Prevalence of Beijing and EAI4-VNM Genotypes among M. tuberculosis Isolates in Northern Vietnam: Sampling Effect, Rural and Urban Disparities
Source: PLoS One. 2012 Sep 24;7(9):e45553. doi: 10.1371/journal.pone.0045553 (PMC3454422; doi:10.1371/journal.pone.0045553)
Supplement: Figure S2 — The collections of M. tuberculosis isolates in the northern Vietnam. (PDF) [file pone.0045553.s002.pdf]

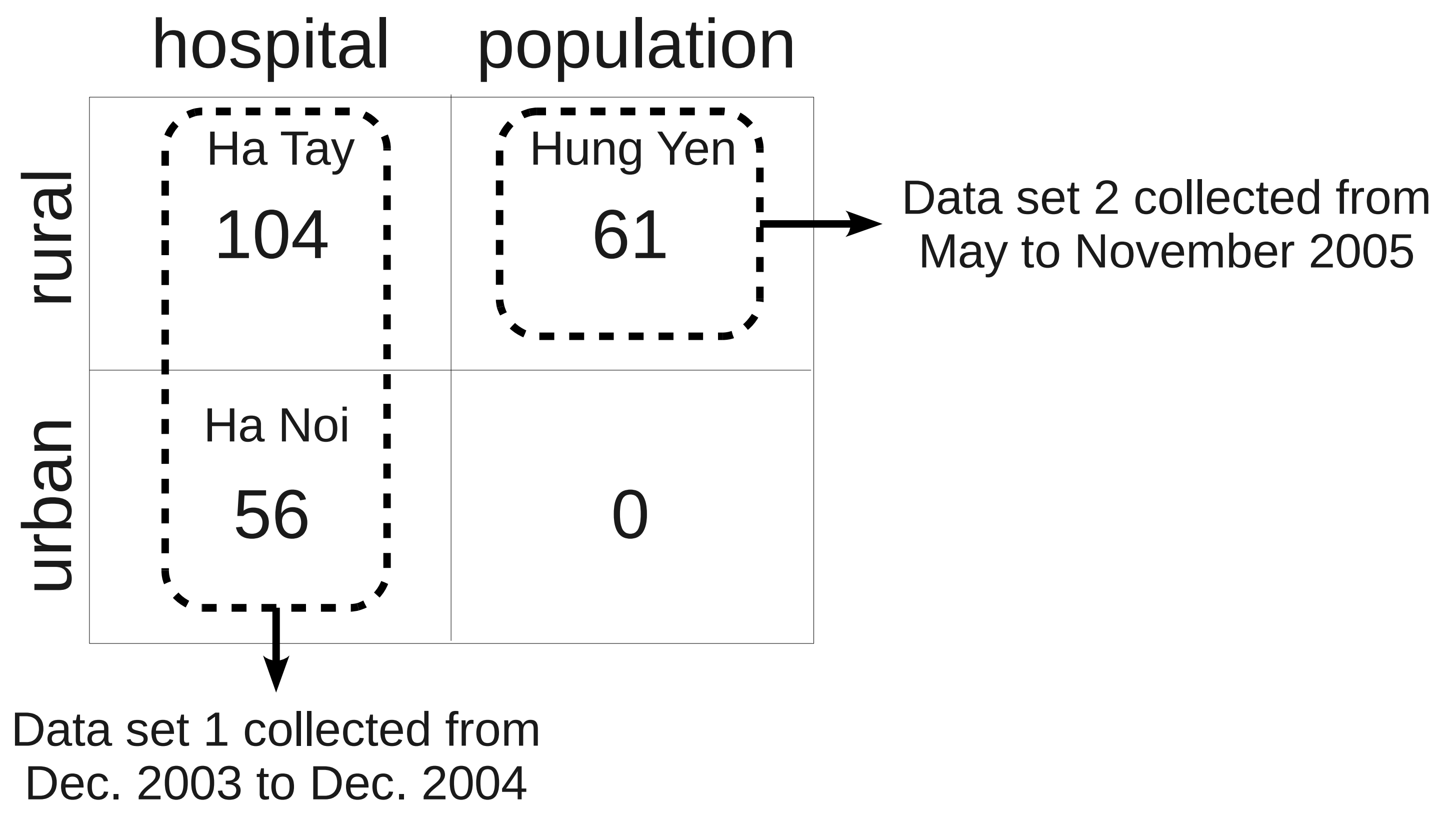

hospital
population
rural
urban
Ha Tay
104
Hung Yen
61
Ha Noi
56
0
Data set 2 collected from
May to November 2005
Data set 1 collected from
Dec. 2003 to Dec. 2004
