# Supplementary material for: High Prevalence of Beijing and EAI4-VNM Genotypes among M. tuberculosis Isolates in Northern Vietnam: Sampling Effect, Rural and Urban Disparities
Source: PLoS One. 2012 Sep 24;7(9):e45553. doi: 10.1371/journal.pone.0045553 (PMC3454422; doi:10.1371/journal.pone.0045553)
Supplement: Table S2 — MIRU types of Beijing, EAI4-VNM strains and the strains other than Beijing and EAI4-VNM from northern Vietnam (separate file). (DOC) [file pone.0045553.s004.doc]

**Supplementary table S2**. MIRU types of Beijing, EAI4-VNM strains and the strains other than Beijing and EAI4-VNM from northern Vietnam.

**A. MIRU types of the Beijing *M. tuberculosis* strains**

| No | 12-MIRU type ^a^ | 12 MIRUs^b^ | |  | No of isolates | | Additional 9MIRUs of clustered isolates^c^ | | No of isolates^d^ |
| --- | --- | --- | --- | --- | --- | --- | --- | --- | --- |
| 1 | MIT17 (M11) | | 223325173533 | | | 17 | | 444434736^e,f^ | 3 |
|  |  | |  | | |  | | 44443245(10)^e^ | 2 |
|  |  | |  | | |  | | 444432459^e,f^ | 1 |
| 2 | MIT84 (M21) | | 223325183533 | | | 1 | |  |  |
| 3 | MIT86 (M01) | | 223325173433 | | | 1 | |  |  |
| 4 | B_Mnvn1 | | 223325173434 | | | 1 | |  |  |
| 5 | B_Mnvn2 | | 223325173434 | | | 1 | |  |  |
| 6 | B_Mnvn3 | | 223325173530 | | | 1 | |  |  |
| 7 | MIT229 | | 224325173533 | | | 1 | |  |  |
| 8 | MIT214 (M19) | | 203325173533 | | | 1 | |  |  |
| 9 | MIT889 | | 223325171533 | | | 2 | | 44432559 | 2 |
| 9 | MIT96 (M47) | | 223325171531 | | | 1 | |  |  |
| 11 | MIT276 (M13) | | 223326171531 | | | 3 | | 44422559 | 3 |
| 12 | B_Mnvn4 | | 223326171b31 | | | 1 | |  |  |
| 13 | MIT166 | | 223225143533 | | | 2 | |  |  |
| 14 | B_Mnvn5 | | 223325143536 | | | 1 | |  |  |
| 15 | B_Mnvn6 | | 223325173323 | | | 1 | |  |  |
| 16 | MIT16(M02) | | 223325153533 | | | 5 | | 444434736 | 2 |
| 17 | B_Mnvn7 | | 223225153333 | | | 1 | |  |  |
| 18 | B_Mnvn8 | | 224225153533 | | | 1 | |  |  |
| 19 | B_Mnvn9 | | 223325153236 | | | 1 | |  |  |
| 20 | MIT83 (M33) | | 223325163533 | | | 18 | | 444432648^f^ | 10 |
| 21 | B_Mnvn10 | | 323325163533 | | | 1 | |  |  |
| 22 | B_Mnvn11 | | 223325163432 | | | 1 | |  |  |
| 23 | B_Mnvn12 | | 223325164534 | | | 1 | |  |  |
| 24 | B_Mnvn13 | | 243327163533 | | | 1 | |  |  |
| 25 | B_Mnvn14 | | 223325163521 | | | 1 | |  |  |
| 26 | B_Mnvn15 | | 2233251a3521 | | | 1 | |  |  |
| 27 | B_Mnvn16 | | 223325183521 | | | 2 | |  |  |
| 28 | B_Mnvn17 | | 223325163543 | | | 1 | |  |  |
| 29 | MIT443 | | 222325163543 | | | 1 | |  |  |
| 30 | MIT104 (M28) | | 222325173543 | | | 9 | | 444432559 | 7 |
| 31 | B_Mnvn17 | | 222315192532 | | | 1 | |  |  |
| 32 | B_Mnvn19 | | 223325112423 | | | 2 | |  |  |
| 33 | B_Mnvn20 | | 224425163421 | | | 1 | |  |  |

**B. MIRU types of the EAI4-VNM *M. tuberculosis* strains**

|  | | | | | | | | | | |
| --- | --- | --- | --- | --- | --- | --- | --- | --- | --- | --- |
| No | 12-MIRU type ^a^ | 12 MIRUs^b^ | |  | No of isolates | | Additional 9MIRUs of clustered isolates^c^ | | No of isolates^d^ | |
| 1 | MIT58 | | 263225223533 | | | 7 | | 247241257^f^ | | 2 |
| 2 | Orphan | | 262225223533 | | | 2 | |  | |  |
| 3 | EAI4_Mnvn1 | | 232225223533 | | | 1 | |  | |  |
| 4 | EAI4_Mnvn2 | | 233225223533 | | | 2 | |  | |  |
| 5 | EAI4_Mnvn3 | | 263225223534 | | | 1 | |  | |  |
| 6 | Orphan | | 263225223433 | | | 2 | |  | |  |
| 7 | EAI4-Mnvn4 | | 263225223443 | | | 1 | |  | |  |
| 8 | EAI4-Mnvn5 | | 263215223433 | | | 1 | |  | |  |
| 9 | EAI4-Mnvn6 | | 265225223533 | | | 1 | |  | |  |
| 9 | EAI4-Mnvn7 | | 265225223543 | | | 1 | |  | |  |
| 11 | EAI4-Mnvn8 | | 265225223532 | | | 3 | | 247241257 | | 2 |
| 12 | EAI4-Mnvn9 | | 265225223542 | | | 1 | |  | |  |
| 13 | Orphan | | 264225223542 | | | 1 | |  | |  |
| 14 | EAI4-Mnvn10 | | 295225223522 | | | 2 | | 246241257 | | 2 |
| 15 | EAI4-Mnvn11 | | 263226223633 | | | 1 | |  | |  |
| 16 | EAI4-Mnvn12 | | 223125223533 | | | 2 | |  | |  |
| 17 | Orphan | | 263225223523 | | | 1 | |  | |  |
| 18 | EAI4-Mnvn13 | | 273325223523 | | | 1 | |  | |  |
| 19 | EAI4-Mnvn14 | | 263224223522 | | | 1 | |  | |  |
| 20 | EAI4-Mnvn15 | | 263224223512 | | | 1 | |  | |  |
| 21 | EAI4-Mnvn16 | | 253225223533 | | | 2 | | 247241257 | | 2 |
| 22 | EAI4-Mnvn17 | | 253225221533 | | | 1 | |  | |  |
| 23 | EAI4-Mnvn18 | | 223225121533 | | | 1 | |  | |  |
| 24 | MIT59 | | 264225223533 | | | 4 | | 247241257 | | 3 |
| 25 | MIT429 | | 274225223533 | | | 1 | |  | |  |
| 26 | EAI4-Mnvn19 | | 224225223733 | | | 1 | |  | |  |
| 27 | EAI4-Mnv20 | | 224235223732 | | | 1 | |  | |  |
| 28 | MIT269 | | 254225223533 | | | 1 | |  | |  |
| 29 | EAI4-Mnvn21 | | 254225223633 | | | 1 | |  | |  |
| 30 | EAI4-Mnvn22 | | 254225221633 | | | 1 | |  | |  |
| 31 | EAI4-Mnvn23 | | 264225221623 | | | 1 | |  | |  |
| 32 | EAI4-Mnvn23 | | 254225223433 | | | 1 | |  | |  |
| 33 | EAI4-Mnvn25 | | 354225223446 | | | 1 | |  | |  |
| 34 | EAI4-Mnvn26 | | 364235222446 | | | 1 | |  | |  |

**C. MIRU types of the *M. tuberculosis* strains other than Beijing and EAI4-VNM**

| No | 12-MIRU type ^a^ | 12 MIRUs^b^ |  | No of isolates | | Additional 9MIRUs of clustered isolates^c^ | | No of isolates^d^ | |
| --- | --- | --- | --- | --- | --- | --- | --- | --- | --- |
| 1 | MIT59 | 264225223533 | | | 2 | | 246241294 | | 2 |
| 2 | MIT81 | 264225223523 | | | 1 | |  | |  |
| 3 | Mnvn1 | 264225223333 | | | 1 | |  | |  |
| 4 | Mnvn2 | 264225223733 | | | 1 | |  | |  |
| 5 | Orphan | 264226223533 | | | 1 | |  | |  |
| 6 | Mnvn3 | 224226223532 | | | 1 | |  | |  |
| 7 | Mnvn4 | 294225223533 | | | 1 | |  | |  |
| 8 | Mnvn5 | 264225223534 | | | 1 | |  | |  |
| 9 | MIT269 | 254225223533 | | | 1 | |  | |  |
| 10 | Mnvn6 | 254226223533 | | | 1 | |  | |  |
| 11 | Mnvn7 | 264225223813 | | | 1 | |  | |  |
| 12 | Orphan | 264225223543 | | | 1 | |  | |  |
| 13 | Mnvn8 | 264225223443 | | | 1 | |  | |  |
| 14 | Mnvn9 | 265225223542 | | | 1 | |  | |  |
| 15 | Mnvn10 | 264225223743 | | | 2 | | 247241257 | | 2 |
| 16 | Mnvn11 | 263225223543 | | | 1 | |  | |  |
| 17 | Mnvn12 | 273225223543 | | | 1 | |  | |  |
| 18 | Mnvn13 | 163225223543 | | | 1 | |  | |  |
| 19 | Mnvn14 | 223225223343 | | | 1 | |  | |  |
| 20 | MIT45 | 225325153323 | | | 1 | |  | |  |
| 21 | Mnvn15 | 225323153333 | | | 1 | |  | |  |
| 22 | Mnvn16 | 222325153423 | | | 1 | |  | |  |
| 23 | Mnvn17 | 222425153223 | | | 2 | | 242232239 | | 2 |
| 24 | MIT743 | 225325163323 | | | 1 | |  | |  |
| 25 | MIT1106 | 226325163423 | | | 1 | |  | |  |
| 26 | MIT40 | 225125113322 | | | 1 | |  | |  |
| 27 | Mnvn18 | 225125111322 | | | 1 | |  | |  |
| 28 | Mnvn19 | 224125114322 | | | 1 | |  | |  |
| 29 | Mnvn20 | 245125113322 | | | 1 | |  | |  |
| 30 | Mnvn21 | 225125112222 | | | 1 | |  | |  |
| 31 | MIT617 | 228225183433 | | | 1 | |  | |  |
| 32 | Mnvn22 | 228225181433 | | | 1 | |  | |  |
| 33 | Mnvn23 | 227225183443 | | | 1 | |  | |  |
| 34 | Mnvn24 | 223225123424 | | | 1 | |  | |  |
| 35 | Mnvn25 | 223225122421 | | | 1 | |  | |  |
| 36 | Mnvn26 | 265325233644 | | | 6 | | 245231267 | | 6 |
| 37 | MIT738 | 224226143321 | | | 1 | |  | |  |
| 38 | Mnvn27 | 242325132422 | | | 1 | |  | |  |
| 39 | Mnvn28 | 123226143337 | | | 1 | |  | |  |
| 40 | MIT715 | 223325163543 | | | 1 | |  | |  |
| 41 | MIT112 | 223325143324 | | | 1 | |  | |  |
| 42 | Mnvn29 | 224325164321 | | | 1 | |  | |  |
| 43 | MIT628 | 126326153227 | | | 1 | |  | |  |
| 44 | Mnvn30 | 253225221633 | | | 1 | |  | |  |
| 45 | Mnvn31 | 263225223632 | | | 1 | |  | |  |

^a^ MIT and Orphan: International type and orphan type identified in SITVITWEB (<http://www.pasteur-guadeloupe.fr:8081/SITVIT_ONLINE/index.jsp>); M: MIRU type previously identified by Mokrousov et al. ; B_Mnvn, EAI4-Mnvn and Mnvn: The Vietnam-Beijing MIRU type, the Vietnam-EAI4-VNM MIRU type and the Vietnam-MIRU type of strains other than Beijing and EAI4-VNM identified in this study.

^b^ The order of the standard 12-MIRU set: MIRU02, MIRU04, MIRU10, MIRU16, MIRU20, MIRU23, MIRU24, MIRU26, MIRU27, MIRU31, MIRU39, MIRU40.

^c^ The order of the 9 additional MIRUs which are used for 15-MIRU typing compared with 12-MIRU typing: Mtub04, ETRC, ETRA, Mtub30, Mtub39, QUB4156, QUB11b, Mtub21, QUB26.

^d^ Number of isolates remained in cluster after 15-MIRU typing.

^e^ Generated by 15-MIRU typing from the MIT17 cluster.

^f^ The genotype remains in the core position after 15-MIRU typing.
